# Causal Analysis of Activity in Social Brain Areas During Human-Agent Conversation

**DOI:** 10.3389/fnrgo.2022.843005

**Published:** 2022-05-17

**Authors:** Caio De Castro Martins, Thierry Chaminade, Marc Cavazza

**Affiliations:** ^1^University of Greenwich, London, United Kingdom; ^2^Institut de Neurosciences de la Timone (INT, UMR7289), Aix-Marseille University-CNRS, Marseille, France; ^3^National Institute of Informatics, Tokyo, Japan

**Keywords:** fMRI, convergent cross mapping, functional connectivity, social interaction, virtual agent

## Abstract

This article investigates the differences in cognitive and neural mechanisms between human-human and human-virtual agent interaction using a dataset recorded in an ecologically realistic environment. We use Convergent Cross Mapping (CCM) to investigate functional connectivity between pairs of regions involved in the framework of social cognitive neuroscience, namely the fusiform gyrus, superior temporal sulcus (STS), temporoparietal junction (TPJ), and the dorsolateral prefrontal cortex (DLPFC)—taken as prefrontal asymmetry. Our approach is a compromise between investigating local activation in specific regions and investigating connectivity networks that may form part of larger networks. In addition to concording with previous studies, our results suggest that the right TPJ is one of the most reliable areas for assessing processes occurring during human-virtual agent interactions, both in a static and dynamic sense.

## 1. Introduction

There has been considerable interest in studying human responses to artificial agents (AA), such as humanoid robots, avatars and chatbots, in various social, and communication contexts. These studies can be classified as investigating the effects of the realism of the agents themselves or taking advantage of the full controllability of AAs to create synthetic environments to investigate how specific aspects affect the human responses. On the other hand, the social cognitive neuroscience theoretical framework of “two-persons neuroscience” (Schilbach et al., 2013) claims that understanding human social cognition requires new experimental paradigms in which natural human-human interactions (HHI) are investigated; it has been proposed that using interactions with AA as control conditions provides relevant comparisons to pursue this goal (Chaminade, 2017).

Actually, there is no clear framework, except the Uncanny Valley hypothesis (Cheetham et al., 2011; Rosenthal-von der Pütten et al., 2019), that describes whether social interactions between a human and an AA are improved when the AA is fully realistic, or whether its artificial nature should be kept visible - and to what extent. Therefore, interactions with AA should be investigated to assess directly whether realism, in terms of human-like appearance (Wiese et al., 2018) nor behavior, should be pursued or whether concepts such as the Uncanny Valley convincingly suggest that such realism should be avoided. Previous study, specifically investigating differences in brain processing when interacting with social robots (Chaminade and Okka, 2013; Wykowska, 2020) yielded contradictory results in terms of the social stance the AA induces in the human interacting partner. Other studies take advantage of the full controllability of virtual agents' behavior to improve the design of complex social or affective neuroscience experiments investigating live interactions. For instance, the ability to finely control the Action Units (AUs) of facial expressions offers controllable and dynamic realism which is neither accessible through the use of video footage or human actors in a laboratory setting (Aranyi et al., 2016), or online control of eye gaze that has been used to investigate joint attention (Schilbach et al., 2010). Yet these studies largely neglect the fact that the artificial nature of AA might itself modify the stance adopted by the human partner (Dennett, 1987). However, few studies directly compared the neuronal and cognitive processes in humans interacting with an AA or a human during free flowing, natural, conversational interactions. In this article, we report results from the analysis of a functional magnetic resonance imaging (fMRI) dataset comprising similar human-human and human-robot natural interactions, to understand how the social ergonomics of one robotic conversational head influence the cognitive processes involved in the interaction. It should be emphasized that, while the artificial nature of the interacting agent is obvious to the participant and an undeniable variable of the experimental set-up, results and their interpretations are limited to this specific artificial agent, in terms of both its appearance and its behavior. In other words, the results reported here need to be further confirmed with AA with different appearances in order to determine what is causally related to the artificial nature of the agent from what results from its specific features.

After reviewing previous study on neuroimaging of human-agent interaction, we describe our fMRI dataset. We then provide an analysis of the data in terms of regional activity focusing on the most relevant regions for the perception of, mentalization toward, and bonding with interacting agents. This analysis is subsequently refined by investigating causal patterns of activation between pairs of these regions where causal connections are believed to play important roles in social interactions given the existing literature, comparing the patterns between the experimental conditions in which the participant interacts with a human and with the robot. We then discuss our findings from the perspective of potential explanatory mechanisms, while also reflecting on the limitations of the analysis and the experimental approach.

### 1.1. Objectives

We use a dataset that was recorded in an ecologically realistic context for dialog so as to investigate the social neuroscience aspects of the interactions. Our goal is to find evidence of differences in neural and cognitive mechanisms between interaction with humans and interaction with an AA, the robotic conversational head from Furhat Robotics in the present case (Al Moubayed et al., 2012). We do not investigate the Uncanny Valley as we do not vary nor mitigate the level of realism of the virtual agent, nor explore users' explicit preferences or human-likeness ratings. Furthermore, such subjective ratings remain very difficult to quantify objectively, one of the reasons the explanatory value of the Uncanny Valley hypothesis can—and should—be debated within a rigorous framework, which is not the objective of the current study. Our purpose is instead to quantify, given one level of human-likeness of an artificial agent, whether cognitive processes differ from the same interaction with a human agent using a specific metric of neural connectivity as an objective dependant variable.

## 2. Previous and Related Study

Cheetham et al. (2011) investigated the Uncanny Valley hypothesis on various perception and categorization tasks involving real human pictures and variably morphed virtual agents, defining, in particular, a Degree Of Humaneness. One low-level, yet relevant finding, is that when studying the response to change in physical qualities between face pairs, activity in the fusiform gyrus (FG) was right-localized for avatar face pairs in contrast to human ones. They also pointed out the uncertainty surrounding the role of image texture vs. geometrical features when categorizing appearance. The FG contains regions responsive to the visual perception of faces, known as the fusiform face area (FFA) (Kanwisher et al., 1997). Here, given the absence of a face “localizer” allowing us to delineate the FFA precisely, we selected an area from the brain parcellation of Fan et al. (2016), which is “blind” to specific functions but is based on functional homogeneity of the voxels included in each area, on the basis of previously reported coordinates of the FFA (Kanwisher et al., 1997).

Rosenthal-von der Pütten et al. (2019) studied the continuous evaluation of virtual agents by humans alongside the Uncanny Valley continuum. They concluded to distinct mechanisms for encoding human-likeness and likeability, the latter being reflected in the activity of the ventromedial prefrontal cortex (VMPFC). They distinguished between the linear encoding of human-likeness in the temporoparietal junction (TPJ) whose activity is not influenced by human-likeness or likeability, vs. nonlinear responses in the dorsomedial prefrontal cortex (DMPFC) and FG underpinning a human-nonhuman distinction. A more detailed analysis would suggest positive human-likeness encoding in the TPJ and negative human-likeness in the FG. However, activity in the TPJ exhibits a negative modulation with human-likeness primarily for nonhuman stimuli but only an average response to human stimuli, suggesting a different response mapping for the two types of stimuli.

In their review of the centrality of social interactions in brain function, Hari et al. (2015) have discussed the role of various networks and regions in different social tasks, including mentalizing. Regions they identified in the mentalizing network include TPJ and DMPFC but also the superior temporal sulcus (STS). They highlight that different subregions of the STS may be tuned to various social stimuli, making it a central hub for social perception. In addition, they suggest, following others (Saxe and Kanwisher, 2003; Van Overwalle, 2009), that the TPJ may play a role in inferring temporary states of other agents, which could be of particular relevance for short-term interactions such as the ones staged in our experiments. Therefore, while the STS, in particular, has not been discussed specifically in relation to interaction with artificial agents, its centrality in social cognitive processes, further confirmed by the integration of multisensory (in particular audition and vision) social signals (Allison et al., 2000; Van Overwalle, 2009), make it an important hub to investigate neural networks involved in social interactions.

Aranyi et al. (2016) have studied the potential for establishing relationships with virtual agents through an fNIRS neurofeedback paradigm controlling the agent's facial expression from the dorsolateral prefrontal cortex (DLPFC) asymmetry intended as a marker of social interest through the approach dimension. The study has discussed the relevance of the DLPFC and VMPFC in human-agent relationships, although it was unclear to which extent the VMPFC signal was contributing to the neurofeedback signal. While DLPFC asymmetry is recognized as a very relevant signal for neurofeedback in view of its controllability, there are less data available on its spontaneous variation during interactions with virtual agents.

Hortensius and Cross (2018) have identified a functional convergence of cognitive factors driving attribution of social characteristics to virtual agents, which draws significantly from the mentalizing network, in particular the DMPFC and TPJ.

Goelman et al. (2019) have studied connectivity during joint attention in human communication using an original experimental design based on a 4-region network concept. The single region (either TPJ or DMPFC) in one brain is matched to three regions in the other brain taken from the VMPFC, PM, STS, and the FFA located in the FG, and the precuneus. Experiments are repeated with the single regions in either the ‘sender' or ‘receiver' brain. Among a set of complex results, the TPJ appeared more involved in the receiving processes; when these were part of the receiving feedback system, the FFA would also appear in pathways with the TPJ in both hemispheres.

Previous study has identified a consistent set of regions of interest (ROIs) during interactions between human users and AA. These regions overlap with traditional findings from social neuroscience regarding human-human communication, yet specific phenomena related to the encoding of human-like appearance in those regions have also been identified, making the corresponding regions even more relevant for our own experiments. Finally, the role and relevance of specific regions cannot be dissociated from the experimental paradigms through which they have been studied. Most previous studies on human-likeness have sought to explore the validity of the Uncanny Valley hypothesis and as such have implemented decision making tasks such as judgments on appearance, identification, or preference; this may in turn have given more prominence to areas associated with such processes, e.g., the VMPFC. One specificity of our study, as well as its originality, rests with the absence of an explicit task, as well as the ecological conditions for observing human-robot interaction.

Based on the above findings, we have, thus, decided to privilege several areas including the STS for its “social hub” role, the TPJ for its role in short-term mentalizing and encoding of human-likeness (Saxe and Kanwisher, 2003), and the FFA for its role in face recognition, and previous reports of differential activation when observing a human or synthetic face (de Borst and de Gelder, 2015). Areas of the PFC have been associated with social relationships, with VMPFC more often activated when preference or human-likeness judgments were requested from the user. DLPFC is primarily of interest *via* DLPFC asymmetry as a marker of approach, a high-level dimension that can serve as a proxy for social engagement; it should be noted, however, that in some cases it may be difficult to distinguish approach from valence. We were also encouraged to investigate lateralization for areas of the temporal lobe given known lateralization of functions, with the left hemisphere bias toward linguistic contents in the STS and TPJ, while the right hemisphere is more systematically found than the left in the mentalizing task (refer to e.g., Saxe and Kanwisher, 2003). We, therefore, conducted separate analyses for each of the two brain hemispheres, for each variable, except for the asymmetry of the DLPFC activity that requires incorporating the signal from both hemispheres.

However, we have not retained other candidate regions mentioned in previous study because our experimental context differed from those in which their role has been investigated. These differences included the absence or presence of explicit human-likeness judgments, self-identification with a virtual avatar, investigation of the role of eye gaze, and the contrast between social interaction and observation. It has been suggested that the mentalizing network was of particular relevance in the latter distinction (Redcay and Schilbach, 2019). For instance, we have not considered the striatum, whose importance has been identified in mutual gaze experiments (Pfeiffer et al., 2014), or the cingulate gyrus involved in self-identification (Ganesh et al., 2012).

## 3. Experiment

We decided to investigate causal relationships between brain regions using Convergent Cross Mapping (CCM) (Sugihara et al., 2012) on a unique corpus including fMRI data acquired during Human-Human and Human-Robot conversational Interactions (respectively HHI and HRI).

### 3.1. Acquisition of the Corpus

The acquisition and processing of this corpus extensively described elsewhere (Rauchbauer et al., 2019), will be presented briefly in this section, focusing on the most relevant aspects for the current analysis. For the entire duration of the experiment, participants were made to believe that they were taking part in a neuromarketing experiment. Upon arrival they were introduced to a “fellow participant” who was actually part of the research team and of the same gender as the experimental subject—experimenter TC for men and master student MB [coauthor in Rauchbauer et al. (2019) for women]. Both were told that one of them (the participant) would be scanned in fMRI while discussing images being designed for an advertising campaign. The question of the putative advertising agency was whether discussing these images was sufficient to figure out the objective of the campaign. There were two campaigns each containing three images, presenting anthropomorphized fruits (fruits disguised as known super-heroes for one campaign, rotten fruits for the second campaign). The cover story was used so that participants were unaware of the real focus of the experiment, the neural bases of social interaction through verbal discussions.

**Figure 1:**
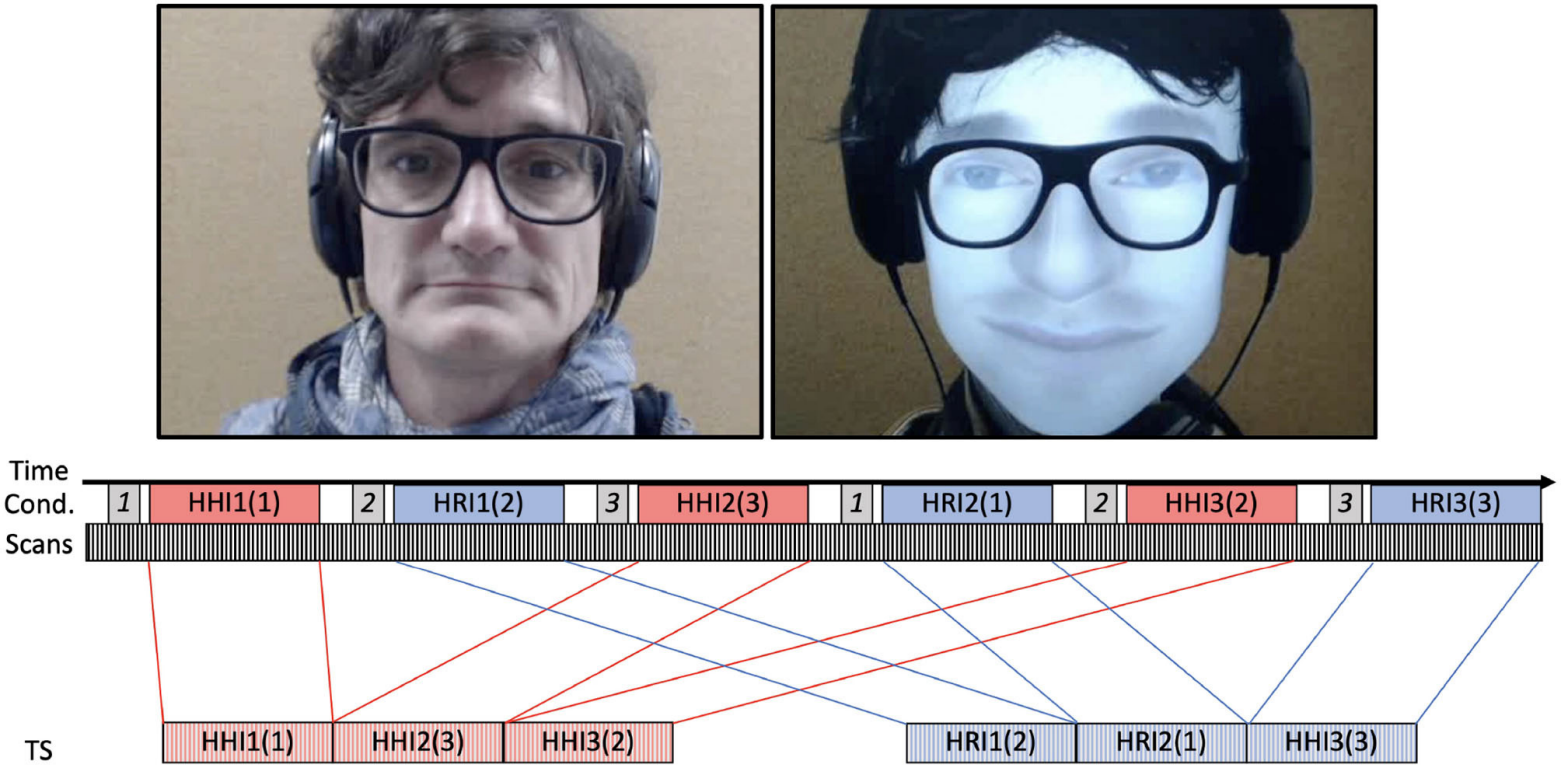
**Top:** Single frames extracted from live video feed projected to scanned participants during HHI (left) and HRI (right). **Bottom:** Example of data acquired for the first session (indexed by time) for every participant. The experimental conditions (Cond) consisted of 6 trials, each starting with the image presented (italic number on gray background) followed by a 1-min discussion with either the human (HHI) or robot (HRI) interlocutor. Continuous acquisition of Blood Oxygen Level-Dependant (BOLD) images (Scans) was subsequently transformed into time series (TS) by concatenating trials with the same interlocutor.

The robot used in the experiment was the original version of a rear-projected speech conversational robotic head from Furhat robotics (Al Moubayed et al., 2012). The facial and voice gender as well as accessories (e.g., a wig) were used to increase the similarity between the human and AA (Figure 1, top). It was controlled by the experimenter with a basic Wizard of Oz implementation (Dahlbäck et al., 1993). The experimenter acting as the human interlocutor selected the robot's response among a set of pre-recorded written responses by pressing virtual buttons on a touchpad. Responses varied from generic (“Yes”, “No” [...]) to very specific (“Maybe it's a campaign to promote local fruits cultivation”). They were stored in written form, and the algorithm controlling the robot created both the lip synchronization projected on the plastic face and the text-to-speech synthesis, yielding a noticeable temporal delay, at the order of few 100 ms, between response selection by the experimenter and the execution of the speech gesture by the robot.

Participants lying supine in the fMRI scanner discussed alternatively with the experimenter and the robot. Importantly, this discussion was unconstrained (i.e., it could take any form and direction) and in real time (i.e., the experimenter heard and responded directly to the participants, whether directly in the HHI conditions or through the conversational robot controlled with the Wizard of Oz interface in the HRI). The audio recorded from both the participants and the external interlocutor were sent to the other agent in real time, meaning there was no imposed turn-taking and that both speakers' occurrences could overlap. The video from the external interlocutor was also projected in real time on a screen located behind the scanner, the participants could see through mirrors located in front of their eyes.

For each participant, in addition to anatomical T1^*^ and T2-weighted images, the BOLD signal (for Blood Oxygen Level-Dependant) was recorded in four sessions of approximately 8 min. Each session consisted of 6 experimental trials (Figure 1, bottom) proceeding as follows: A picture appears for 8.3 s, followed by a 3-s pause with a gray fixation cross on a black background. A 1-min real-time discussion then took place, alternating between the human and the artificial interlocutor. During this conversation, in addition to hearing each other, the participant also looks at a live video stream of the interlocutor, human, or robot. In total, 12 1-min conversations with each of the human and robot interlocutors were recorded for each participant.

### 3.2. Processing of the fMRI Data

The analysis of the MRI data is identical to the one in Rauchbauer et al. (2019) and includes twenty-four participants of the final corpus from whom data from the four sessions are available (17 women, μ = 26.76 years, σ = 7.96). All were native French speakers, right-handed with no history of neurological or psychiatric disorders. Preprocessing, described in detail in Rauchbauer et al. (2019), followed standards of *SPM* procedures (Friston, 2007) including slice timing, unwarping for inhomogeneities of the MRI magnetic field, realignment, coregistration with the anatomical image acquired during the same scanning event, segmentation of the anatomical image, coregistration of the segmented images for the 24 participants following *DARTEL* procedure (Ashburner, 2007), and normalization of preprocessed functional time series as well as anatomical images into MNI-space using tensors calculated with the *DARTEL* procedure. Importantly, given that speaking during fMRI acquisition was feared to induce brain movement, a procedure to detect important head movements (*ART* for Artifact Detection Tool, which measures the displacement of the participants' head in the magnet and reports images exceeding a threshold) was used and confirmed that none of the participants exceeded the usual threshold. Nuisance regressors were calculated using the Translational Algorithms for Psychiatry-Advancing Science (*TAPAS*, Frässle et al., 2021) toolbox, which calculates variables able to explain variance due to participants' movements, recorded physiological data (heartbeat and respiration, particularly important given the correspondence between speech and these physiological changes) and global fluctuations in the gray matter, white matter, and cerebrospinal fluid.

At the single participant level, a general linear model (glm) was estimated by the Statistical Parametric Mapping (*SPM12*- toolbox; Friston, 2007) with three conditions of interest (image presentation, HHI and HRI) and fifty-six nuisance regressors. The beta maps estimated, in each participant and session, for the two conditions of interest, HHI and HRI, were entered in a second-level, random effect, whole brain glm analysis (*SPM*; Friston, 2007). The conjunction of the main effect of HHI and HRI is reported with a family-wise error correction of *p* < 0.05 at the voxel-level, while the two contrasts two reciprocal contrasts between HHI and HRI are reported with the less stringent family-wise error correction of *p* < 0.05 at the cluster-level.

### 3.3. ROIs Analysis

Regions of interest were selected on the basis of functional landmarks, either from a global parcellation of the cortex using anatomical and functional connectivity (Brainnetome, Fan et al., 2016) or a parcellation based on large scale functionally connected networks associated with specific functions identified with independent component analysis and implemented in the toolbox *conn* (Whitfield-Gabrieli and Nieto-Castanon, 2012) (“Networks” parcellation). Beta estimates for the two conditions of interest, HHI and HRI, were extracted using the *SPM* toolbox *MarsBar*, and their effect was analyzed in R (R Core Team, 2020) using linear models implemented in the *lme4* package, using Subjects as a random variable package. Estimated marginal means of these betas were calculated using package *lsmeans* following the ANOVA calculated with the *lmertest*. It should be noted that, because of the long duration of trials and in the absence of precisely timed events, we use beta estimates instead of percent signal change to analyze the contribution of experimental conditions HHI and HRI to changes in the BOLD signal over 1-min trials.

**Figure 2:**
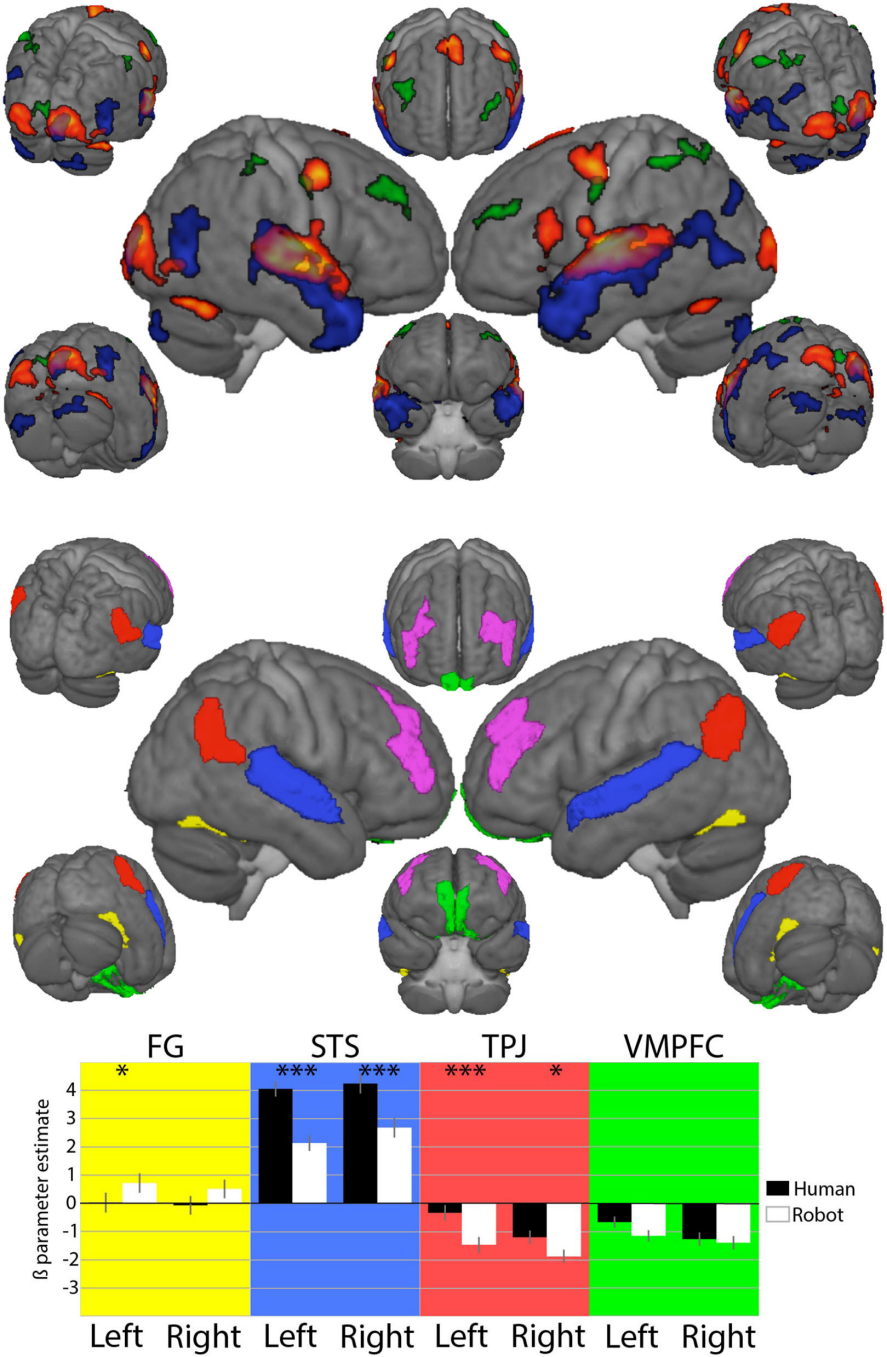
**Top:** On the left, results from the *SPM* second-level analysis, showing the conjunction between conversations with the two agents on a hot scale, and results from the comparison of HHI vs. HRI in blue and HRI vs. HHI in green and on the right, the regions of interest (ROIs) used for the analysis (details in the main text). **Bottom:** Results from generalized linear model analysis of beta estimates extracted from the ROIs. Significance of comparisons ***p < 0.001 and *p < 0.05 is indicated above Human and Robot parameter estimates.

Time series were extracted from *conn* toolbox processing after importing *SPM* first-level analysis, benefiting from *conn* optimized preprocessing of time series and the possibility of using any parcellation of the brain into ROIs. Eventually, several approaches were used to define the different region ROIs based on the hypothesis underlying its definition. We strongly relied on the Brainnetome parcellation as it offers a fine-grained parcellation in which the regions are defined by connectivity homogeneity, both at the effective and functional levels, which ensures that voxels included in the regions are involved in the same process. In the absence of strong hypotheses about the exact position of the ROIs, we designed larger areas by joining together adjacent regions in the cortical areas considered. The final ROIs can be seen in Figure 2).

We joined three adjacent areas covering the posterior part of the temporal sulcus to form the STSROI. The FG was designed by joining the anterior and posterior fusiform regions, and the VMPFC by joining the two most ventral medial parts of the prefrontal cortex, representing the VMPFC. Eventually, the frontal region of interest used to investigate prefrontal asymmetry of brain activity was the most difficult to define; both on the basis of its anatomical location (Aranyi et al., 2016) and functional involvement in attention oriented to external events, the frontal region of the fronto-parietal attention network from the Networks parcellation was chosen as DLPFC ROI instead of one (or a specific junction of several) prefrontal area(s) from the Brainnetome parcellation; DLPFC asymmetry was calculated by subtracting the BOLD signal of the right DLPFC from the left. Continuous time series covering each of the four sessions for the 24 participants were extracted for the two conditions of interest (HHI and HRI).

## 4. Causal Analysis Methodology

We focus on differences in connectivity between pairs of key regions involved in the framework of social cognitive neuroscience, guided by previous research and findings on the role of these regions, their dynamics, and previous connectivity studies. We investigate the existence of a causal connection, the magnitude and direction of causal propagation, and the most appropriate time lags to determine causality. Our approach can, thus, be seen as a compromise between research directly comparing activation in specific regions (such as obviously the FG), and research trying to uncover comparative connectivity networks for the two communication conditions. We posit that investigating connectivity between specific region pairs that may form part of larger networks should still shed light on candidate mechanisms. Not only this may constitute a preliminary step in the future search for more integrated networks, but it is unclear whether we could have started with sufficient hypotheses for the validation of a full-fledged network, nor if data from our experiments are sufficient, quantitatively and qualitatively, for such an endeavor. Previous study on connectivity networks in human vs. humanlike interaction has uncovered networks of limited scale and in a slightly different context of decision making (Rosenthal-von der Pütten et al., 2019).

### 4.1. Analytical Approach

There is a wide range of tools to study the statistical dependencies between two or more neural systems, where these dependencies can be undirected (e.g., correlation) or directed (e.g, Granger Causality and Transfer Entropy; Granger, 1969; Schreiber, 2000). Granger Causality (GC) is a widely used framework for functional connectivity analysis. It uses predictability to model the coupling between time series variables. In its simplest form, GC can be reduced to fitting a linear vector autoregressive (VAR) model. The framework can easily be adjusted to accommodate for other variations of GC such as partial and conditional GC. Furthermore, the spectral decomposition of VAR models gives rise to spectral measures of GC (e.g., Geweke-Granger-causality, directed transfer function, and partial directed coherence; Geweke, 1982; Kaminski and Blinowska, 1991; Baccalá and Sameshima, 2001).

The framework provided by GC is a simple and practical tool for identifying directed functional interactions from time series data. However, GC is limited to cases where the assumptions of VAR modeling are satisfied—weakly stationary linear stochastic process. A fundamental property of linear systems is separability—meaning that causal factors can be removed from effects. Separability is a key requirement for GC; when this requirement is not satisfied, it can lead to spurious results.

In neuroscience, specifically in the context of fMRI time series, the use of GC has been highly controversial as fMRI BOLD responses are convolved with a hemodynamic response function (HRF), thus making them a delayed and indirect measure of the underlying neural processes. However, Seth et al. (2013) showed that, as the HRF acts as a filter, GC is invariant to the HRF convolution, but this invariance is constrained to a fast-sampling rate and low measurement noise. The reader is referred to Cekic et al. (2018) for a more detailed discussion on the topic.

Given the theoretical and practical limitations of GC in the context of fMRI time series. We take a nonlinear data-driven approach, to study pairwise “dynamic” connectivity between ROIs. Our approach makes use of a recent method originally developed to study complex ecological systems (Sugihara et al., 2012) but has also been applied to the dynamics of neuroimaging data (Tajima et al., 2015; Wismüller et al., 2015; Natsukawa and Koyamada, 2017; DSouza et al., 2018; Schiecke et al., 2019; Chowdhury et al., 2020) - CCM.

Convergent Cross Mapping is a novel approach to studying the coupling between time series, it is a type of empirical dynamic modeling (EDM). EDMs are non-parametric frameworks for modeling nonlinear dynamic systems – it is based on the mathematical theory of reconstructing attractor manifolds from time series data (Takens, 1981). EDMs are an alternative and highly flexible approach to using explicit equations since these equations can be impractical when the exact mechanisms are unknown or too complex to be characterized with existing datasets. Motivation for this approach is exemplified by studies that have shown that fMRI time series exhibits nonlinear dynamic behavior (Gautama et al., 2003; Gultepe and He, 2013; Lombardi et al., 2014; Minati et al., 2015).

In contrast to the Granger framework which is aimed at purely stochastic systems that exhibits linear dynamics, CCM addresses cases not covered by Granger which involves nonlinear dynamic systems (Tsonis et al., 2018). Although GC can be used for detecting interactions between strongly coupled variables in nonlinear systems, as noted by Granger (1969), GC is not suitable for dynamic systems with weak to moderate coupling. In contrast, CCM also addresses non-separable systems, with weak to moderate coupling, and is able to distinguish causal interactions from the effects of shared driving variables (Sugihara et al., 2012).

### 4.2. Implementation of the Analysis

#### 4.2.1. Convergent Cross Mapping

In dynamical systems theory, time series variables are causally linked if they originate from the same dynamic system—they share a common attractor manifold. Furthermore, time series are thought of as sequential projections of the motion on an attractor; information about the behavior is encoded in the temporal ordering of the time series. We can reconstruct a shadow version of the original manifold **M** using lagged versions of a time series **x** = *x*(*t*) = {*x*_*t*_; *t* = 1...*L*}. If sufficient lags are used, the reconstructed Manifold **M_x_** preserves essential mathematical properties of the original system, which means that reconstructed states will map one-to-one onto actual system states, and nearby points in the reconstruction will correspond to similar system states. Multiple reconstructions of the manifold not only map one-to-one onto the original system but also onto each other. This suggests that we can test whether the two variables interact in the same system (and are thus causally related), by testing for mapping between their corresponding reconstructed states.

Convergent Cross Mapping uses this idea to test for causation by measuring the extent to which the historical record of **y** values can reliably estimate states of **x**. This is done by seeing whether there is a correspondence between the libraries of nearby points in the attractor manifold reconstructed from **x** (**M_x_**) to that reconstructed from **y** (**M_y_**). **M_x_** is defined as the set of vectors **X** = *X*(*t*) = < *x*(*t*), *x*(*t*−τ), *x*(*t*−2τ), ..., *x*(*t*−(*E*−1)τ)> for *t* = 1+(*E*−1)τ to *t* = *L*. Where *E* corresponds to the embedding dimension (the number of lags of *x*(*t*)) and τ is the time lag between successive dimensions.

Furthermore, because the causal interaction between two signals may not be instantaneous but delayed over a certain time interval (*l*), our implementation of CCM explicitly considers different lags for cross-mapping. This also helps to distinguish between bidirectional causality and strong unidirectional causality that leads to synchrony (Ye et al., 2015).

To obtain a cross-mapped estimate of **y** = *y*(*t*+*l*) = {*y*_*t*+*l*_}, denoted as ŷ(*t*+*l*)|**M_x_**, we locate the contemporaneous lagged-coordinate vector on **M_x_**, and find its *E*+1 nearest neighbors. The time indices of the *E*+1 nearest neighbors (*t*_1_, ..., *t*_*E*+1_ ; from closest to farthest) of **X** are then used to identify nearest neighbors of *y*(*t*+*l*) and obtain an estimate ŷ(*t*+*l*)|**M_x_** from a locally weighted mean of the *E*+1, *y*(*t*_*i*_+*l*) values.


(1) $$\hat{y}(t+l)|M_{x}=\sum_{i=1}^{E+1}w_{i}y(t_{i}+l)$$

where *w*_*i*_ is a weighting based on the distance between *X*(*t*) and its *i*th nearest neighbor on **M_x_** and *y*(*t*_*i*_+*l*) are the contemporaneous values of *y*(*t*+*l*). The weights are determined by a softmax function such that the first nearest neighbor has the highest weight.


(2) $$\begin{array}{r} w_{i}=\frac{\exp\left(-|X\left(t\right)-X\left(t_{i}\right){|}^{2}/|X\left(t\right)-X\left(t_{1}\right){|}^{2}\right)}{\sum_{j=1}^{E+1}\exp\left(-|X\left(t\right)-X\left(t_{j}\right){|}^{2}/|X\left(t\right)-X\left(t_{1}\right){|}^{2}\right)} \end{array}$$

Additionally, *l* is the lag being considered. Negative values of *l* < 0 correspond to estimating the past values of **y** using the reconstructed states of **x**. This suggests that the dynamical signal appears first in **y** and later in **x** and is consistent with **y** causing **x** . If there is no causation in the reverse direction (i.e., **x** does not cause **y**), then the reconstructed states of **y** should best predict future values of **x** and we would expect higher cross-mapping skill in the opposite direction – i.e., *l* > 0. Thus, this “asynchrony” reflecting the time lag in the response can be used to (1) identify time delays in causation, and (2) distinguish between bidirectional causality and generalized synchrony when there is a detectable lag in the response time between causes and effects.

Counter intuitively to Granger, if variable **y** is influencing **x**, then causality is established if the historical record of the affected variable **x** can reliably estimate states of the causal variable **y**. This is quantified by calculating the correlation coefficient ρ between the predicted ŷ(*t*+*l*)|**M_x_** and observed *y*(*t*+*l*).

To distinguishes causation from simple correlation, CCM relies on convergence^1^. That is, the correlation increases with the length of the time series. The relative level to which the correlation converges can be viewed as an estimator of the strength of the causal link. With more data, the underlying attractor manifold becomes denser, and nearest neighbors get closer, resulting in declining estimation error.

#### 4.2.2. Data Preparation

One of the common issues faced in statistical analyses is the sample size. In state space reconstruction methods such as CCM, that equates to attractor dimensionality, as the amount of data required for reconstruction depends on the dimension of the attractor. Though CCM does not concern itself with the dimension of the attractor (*d*), it relates to the embedding dimension of the reconstructed manifold (*E*) through the Whitney embedding theorem (*E* ≤ 2*d*+1). DSouza et al. (2018) showed the deleterious effect that a high repetition time (TR) has on attractor reconstruction. Following the example of McFarlin et al. (2013) who reported improvements in connectivity analysis on up-sampled fMRI data and the nature of the experiment^2^, we re-sampled our BOLD time series data from ≈0.8 *Hz* (50 time points) to ≈3.3 *Hz* (200 time points) using MATLAB's uniformly sampled signal resample function in signal processing toolbox (MathWorks Team, 2021).

#### 4.2.3. Pairwise CCM Analyses

**Figure 3:**
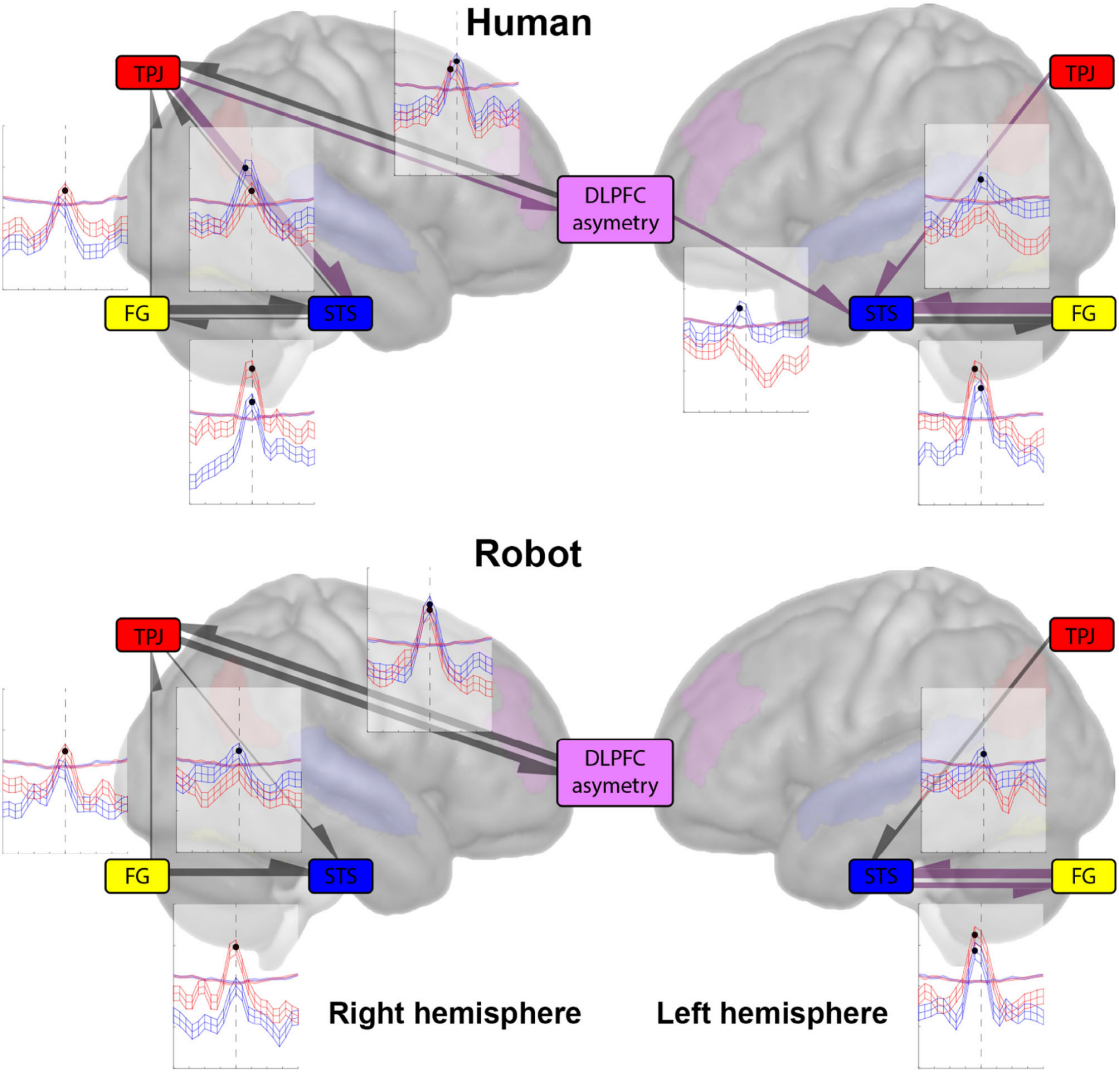
The plots show the mean and standard error cross-mapping skill (ρ) (of the largest library size used for cross-mapping; 200) as a function of cross-mapping lag, across all humans **(top)** and robot **(bottom)** trials (288 each) for the left and right hemispheres. The shaded regions show the mean and standard error cross-mapping skill for the null distribution across all humans/robot trials. The largest CCM skill is depicted by a solid circle marker. The arrows from one region to another represent the direction of causation. The width of the directional arrow is proportional to the distance between the observed (ρ) and the null distribution (ρ)—signifying the strength of causation. The color of the directional arrow represents the delay in causation; the black arrows show an immediate effect from one region onto another (0 lag), while the purple arrows show a delayed effect from one region onto another (–2 lag).

Pairwise connectivity analysis was performed using CCM with the re-sampled BOLD time series, as shown in Figure 3. The analysis was performed for all pairs of ROI, for all trials (576 trials, 288 humans, and 288 robots). To find the optimal choice of reconstruction parameters (namely the embedding dimension *E*, τ was set to 1), we used (Sugihara and May, 1990) *simplex forecasting* method to evaluate the prediction skill for various choices of *E* (1...10), the lowest *E* with the highest forecasting skill was chosen.

To evaluate the appropriateness of the use of CCM, we investigated the nonlinear dynamics of the data using the Sugihara (1994) *s-map* method. The parameters used for this method (namely *E*), were defined as the optimal parameters identified using the *simplex* method. The results of the analysis showed that all ROI exhibited some nonlinear dynamics—confirming that CCM can be used to study pairwise connectivity between regions.

Convergent Cross Mapping analysis constituted of performing CCM at every lag (−20...20, by 2) using the largest library size (200; the whole time series) for every pair of ROI. Similarly, to evaluate the significance of cross-mapping, surrogate analysis^3^ was performed at every lag, and 95% quantile was pulled out of a sample of 100 surrogate cross-mapping.

## 5. Results

### 5.1. General Linear Model Analyses

Whole brain results from the contrasts HHI and HRI (at p < 0.05 FWE-corrected at the cluster level) indicate a shift from temporal and ventral parietal regions for humans to dorsal frontal and parietal regions for the robot contrast as in Rauchbauer et al. (2019). Analyses detail how the experimental conditions impact the response of regions later used in the CCM analysis. There was a significant effect of the agent in the STS [Left: *t*_(1, 167)_ = 15.11, *p* < 0.001; Right: *t*_(1, 167)_) = 12.16, *p* < 0.001], TPJ [Left: *t*_(1, 167)_ = –5.28, *p* < 0.001; Right: *t*_(1, 167)_ = –3.04, *p* = 0.003], but not in the VMPFC (*p* = 0.018 and *p* = 0.531 in the left and right hemispheres respectively). The asymmetry of DLPFC activity is used in the CCM analysis, so that, in contrast to other regions, the difference of activity between the hemispheres is a relevant factor. Thus, the effect of laterality (left *vs* right hemisphere) was computed together with the effect of the Agent in the case of the DLPFC region. In this analysis, the hemisphere of the DLPFC ROI (right vs. left) significantly modifies the activity [*t*_(1, 357)_ = –5.801, *p* < 0.001] with increased estimates for the left hemisphere, but the effect of the agent [*t*_(1, 357)_ = 1.296, *p* = 0.20] and the interaction between hemisphere and agent [*t*_(1, 357)_ = 0.297, *p* = 0.767] are not significant.

### 5.2. CCM Results: Post Central Sulcus Hypothesis

Figure 3 shows the results of CCM cross-mapping as a function of lag for all pairwise analyses. Starting with the left hemisphere, we see that there is unidirectional causation from the TPJ to the STS in both human (mean ρ = 0.1825, lag = −2 ≈ 602.5 ms) and robot (mean ρ = 0.1693, lag = 0) cases, the −2 lag in the human case, is suggestive that the TPJ is acting on the STS with a slight delay compared to the instantaneous effect it has in the robot case. Effects from STS to TPJ are not above the significance threshold set by the null distribution (α = 0.05). In the case of STS and FG, the results show a bidirectional causal relationship between the regions, with FG affecting the STS with a slight lag in both human (mean ρ = 0.2153, lag = −2 ≈ 602.5 ms) and robot (mean ρ = 0.2125, lag = −2 ≈ 602.5 ms) cases. Whereas the STS appears to have a more instantaneous effect on FG in the human (mean ρ = 0.1920, lag = 0) than in the robot (mean ρ = 0.1935, lag = −2 ≈ 602.5 ms) case.

For the right hemisphere, we have a slight change in dynamics compared to the left for the TPJ and STS. Here, the results show a significant cross-mapping in both directions in the human case but the direction in the robot case remains unchanged (mean ρ = 0.1726, lag = 0). For the human case, we have the results being suggestive of a slight delay from the TPJ to the STS (mean ρ = 0.2007, lag = −2 ≈ 602.5 ms) but an immediate effect of the STS on the TPJ (mean ρ = 0.1722, lag = 0). In the right hemisphere, we also have a significant causation from the FG to the TPJ in both human (mean ρ = 0.1707, lag = 0) and robot (mean ρ = 0.1738, lag = 0) cases. For the FG and STS, we have a bidirectional causal relationship, with both FG (mean ρ = 0.2148, lag = 0) and STS (mean ρ = 0.1744, lag = 0) having an instantaneous effect on each other. Whereas, in the robot case, we only have a unidirectional causal relationship from the FG to the STS (mean ρ = 0.1981, lag = 0).

### 5.3. CCM Results: Post to Precentral Sulcus Hypothesis

From Figure 3, in the right hemisphere, we have a bidirectional causal relationship between the DLPFC asymmetry and the TPJ, with the TPJ having a slight lag in causation in the human case (mean ρ = 0.1793, lag = −2 ≈ 602.5 ms), compared to the robot case (mean ρ = 0.1984, lag = 0), while it acts with the same lag on the TPJ in both the human (mean ρ = 0.1893, lag = 0) and robot (mean ρ = 0.2049, lag = 0) cases.

In the left hemisphere, we see no dynamic causal relationship between the DLPFC asymmetry and the FG, nor between the DLPFC asymmetry and the TPJ in either the human nor the robot case. The only significant causation is from the DLPFC asymmetry to the STS (mean ρ = 0.1765, lag = −2 ≈ 602.5 ms) in the human case. The absence of causation between the DLPFC asymmetry and the FG (and STS), indicates that the effects are lateralized.

## 6. Discussion

### 6.1. Localized Brain Activity

Concerning auditive and visual association areas, both whole-brain and region-of-interest analyses converge; there is an absence of FG contribution in both conditions, with no changes between them, while the STS is strongly involved in both conditions—more so in the HHI than in HRI. In the TPJ there are effects in both the agents, with increased response for the human reflected in the blue clusters, visible in the whole brain analysis, with stronger responses in the left hemisphere. Finally, increased response in HRI compared to HHI in the ROI analysis corroborates the bilateral green clusters found in the DLPFC in the whole brain analysis, as well as an asymmetry with increased response in the left compared to the right hemisphere not clearly visible on the renderings of the whole brain analysis.

The absence of an overall response or effect of conditions in the VMPFC is found in both analyses. This region was neither associated with any significant effect, nor any causal analysis (CCM) interpreted in the next section. It is possible that this area is related to decision making in situations that are investigated in decision making experiments, e.g., those inspired by game theory. This is very remote from the current situation, in which the decision relates to how to interact with a human or artificial agent, and events are very long (1-min trials). It is, therefore, possible that brain states are relatively long, while changes implying the VMPFC are related to faster decision making.

Altogether, the analysis using ROIs based on our hypotheses reproduce roughly observations made at the cortical level in the whole brain analysis and already reported in Rauchbauer et al. (2019), yet few conclusions can be drawn concerning the causal relationships between the experimental conditions and these results. Indeed, differences in participants' behaviors, as they were unconstrained, can explain differences in local brain activity without requiring reference to the nature of the agent. One simple example is the STS region: while both conditions involved processing produced and perceived speech, it is possible that the increase of speech processed, and not the nature of the agent, explains the increased response of the STS region for the human compared to the robot conversations. However, the CCM analysis used here is immune to this criticism as it takes into account causal relationships through time, irrespective of the underlying behaviors. This increases its ability to improve the understanding of causal relationships between pairs of regions in the two aspects of our hypotheses, namely, how speech and visual sensory information are integrated within a social process depending on the artificial or human nature of the interlocutor, and which of these pieces of information influences prefrontal processes involved in the social competence of the interaction encoded in the prefrontal cortex.

### 6.2. Interpretation of the CCM Results

First generic comment is that all the significant effects (largest (ρ)'s deviation from surrogate) we found are equal or close to a lag of 0 (0 or –2, approximately 600 ms). As stated earlier, both the length of the processes under investigation (1 min) and the unconstrained nature of the task do not allow us to look at frequent reproducible events but at continuous processes for specific timing, so that there are no time relationships between single trials. The most important finding is the implication of the right TPJ in both the posterior sensory and posterior-to-anterior sensory to social cognitive processes. While we were primarily concerned with the comparison of cross-mapping between STS and TPJ, the difference between results in the left and right hemispheres is more striking. As shown in Figure 3, only the FG-STS relationships are significant for both hemispheres and both agents, not reaching significance only from STS to FG in the right hemisphere for the robot. Results also show a unidirectional influence of the TPJ on the STS, with a lag of –2 for the human, absent for the robot. Altogether, these results tend to confirm an important role of the STS in the integration of sensory information pertaining to social cognition, while this role was hypothesized for the TPJ region instead. In particular, the direct influence of the DLPFC asymmetry on the left STS activity in humans was not anticipated and further confirms the role of the left STS for integrating multiple sources of social information, from sensory (i.e., FG) but also contextual (i.e., DLPFC asymmetry) information in the current setting, that could be explained by the dominance of left hemisphere involvement in the verbal interactions used for the interaction. In the CCM between post and precentral regions, the two reciprocal CCM between the right TPJ and DLPFC asymmetry were significant for both agents, while most of the CCM between STS and DLPFC asymmetry as well as between the MOFC and all postcentral regions were below surrogate. When users interact with the robot face, we observe a strong, bidirectional, and almost immediate (i.e., zero lag) connectivity between DLPFC asymmetry and TPJ. Such a finding is challenging to interpret in terms of cognitive mechanisms should we follow our initial intuition of using DLPFC asymmetry as a proxy for engagement (*via* the approach dimension), in particular, comparing it to the connectivity between the same regions during interaction with a human face. However, DLPFC asymmetry has also been associated with various situations involving stress (Parent et al., 2020), cognitive workload, and even communication apprehension (Beatty et al., 2011). We would, thus, tend to favor, albeit cautiously, the latter explanation and consider that the observed phenomena correspond to the difficulty for users to integrate social signals in the TPJ for the robot case. Altogether, this supports a central role for the right TPJ to both integrate sensory signals from face and voice processing during a natural linguistic interaction (Campanella and Belin, 2007).

### 6.3. Relations to Previous Study and Findings

Our CCM analysis of functional connectivity between selected **ROIs** was grounded on the hypothesis of a network involving STS, FG, and TPJ in the integration of sensory signals during natural conversations. Previous study (Rosenthal-von der Pütten et al., 2019) on user preferences depending on agent human-likeness had identified a network involving FG, TPJ, DMPFC, and VMPFC during choice tasks; and FG, TPJ, and DMPFC during agents' ratings. They concluded that areas implicated in valuations of human-likeness, likability, and subjects' decisions interacted functionally during decision making. As discussed in the previous section, the involvement of VMPFC might be precisely explained by the decision making component, while our experiments are essentially task free. On the other hand, the greater role of STS can be explained by the truly interactive nature of the experimental procedure, as opposed to, e.g., judgment tasks involving static pictures.

The stronger connection we observed in the right hemisphere between FG and TPJ appears consistent with previous findings on the role of right TPJ in the evaluation of human-likeness (Jack et al., 2013). However, the limited difference in connectivity between the human and robot case is a reminder of the similar activation of the FG in both contexts; although this finding is counter-intuitive, it could find its explanation in the use of mapped video on the robot's face, if one subscribed to the hypothesis that texture plays a determinant role in face analysis (Seyama and Nagayama, 2009; Cheetham et al., 2011).

The most significant differences in connectivity between the human and the robot case are observed for the link between STS and TPJ: one possible interpretation in this context is that perspective taking (TPJ) is dependent on the social perception but more challenging in the robot case. Altogether, the right TPJ appears to be one of the most reliable areas to assess processes happening during human-AA interactions, not only from static but also from a dynamic point of view.

## 7. Conclusion

We investigated how relations between brain regions hypothesized to be involved in social interactions are modulated by the nature, real or artificial, of an interacting agent during a natural conversation. We used a causal approach based on CCM in an attempt to uncover potential differences not just in regions' activation, but in candidate integration mechanisms. The approach allowed the identification of processes that are common to the two conditions, in particular, the influence of the FG on the STS region bilaterally, as well as on the right TPJ. Other results differed depending on the nature of the agent, such as the convergence of influences from the prefrontal asymmetry and left TPJ and FG on the left STS region that were all significant only for the human partner. A stronger reciprocal influence between the right TPJ and dorsolateral asymmetry for the robot than for the human agent was attributed, not to the approach dimension within a complete communication loop, but other activation mechanisms of the DLPFC, reflecting difficulties with processing the robotic agent's information. Overall, our results confirm the role of the left STS in combining different sources of information related to conversational exchanges during unconstrained, ecological interaction,while the asymmetry in prefrontal activity, that did not differ between the two agents despite our hypotheses, was strongly influenced by an area involved in attribution of mental states. Though further study, involving the finer definition of **ROIs** including some that were not considered here, such as the DMPFC, is required to better describe the dynamics of information processing in HHI and HRI, the present results are consistent with findings in previous literature, and also comfort the use of CCM to investigate complex inter-area interactions.

## Data Availability Statement

The raw data supporting the conclusions of this article will be made available by the authors, without undue reservation.

## Ethics Statement

Written informed consent was obtained from the individual(s) for the publication of any potentially identifiable images or data included in this article.

## Author Contributions

CD, TC, and MC have made direct and substantial intellectual contributions to the article and approved it for publication. CD implemented CCM software, conducted the connectivity analysis, and contributed to the writing of the paper. TC provided the neuroimaging data and contributed to the writing of the paper. MC contributed to the methodology, interpretation, and to the writing of the paper. All authors contributed to the article and approved the submitted version.

## Funding

The corpus used in the analysis presented in this paper was acquired with support from grants ANR-11-LABX-0036 (BLRI), ANR-16-CONV-0002 (ILCB), and AAP-ID-17-46-170301-11.1 by the Aix-Marseille UniversitÃl' Excellence Initiative (A*MIDEX).

## Conflict of Interest

The authors declare that the research was conducted in the absence of any commercial or financial relationships that could be construed as a potential conflict of interest.

## Publisher's Note

All claims expressed in this article are solely those of the authors and do not necessarily represent those of their affiliated organizations, or those of the publisher, the editors and the reviewers. Any product that may be evaluated in this article, or claim that may be made by its manufacturer, is not guaranteed or endorsed by the publisher.

## Footnotes

^1^Convergence is a key property for inferring causality, it is limited by observational error, process noise, and time series length. CCM is established by predictability that increases with library size – i.e., the amount of data that is used for the reconstruction of the shadow manifold. Failure to account for convergence can lead to spurious results, as cross-mapping that is limited to the statistical association between variables will generally not improve with an increase in library size. Since convergence is a key determinant for CCM, there is no consensus in the literature on values of ρ that are associated with causality. The reader is referred to Sugihara et al. (2012), Ye et al. (2015), Nakayama et al. (2018), and Ye et al. (v075) for similar levels of cross-mapping as reported in this article.

^2^Given the ecological nature of the conversation task, the pace of the experiment was not as demanding as in most fMRI studies in which the objective is to accumulate as many trials as possible in a given time. For this reason, the brain responses are likely to be best modeled as a succession of steady-states rather than rapid events, and the up-sampling of the time series yields benefits without the cost of losing sharp transitions between events.

^3^The surrogate analysis is implemented as follows: for the effect of **y** on **x**, we cross map from **x** to **y**, therefore, to understand whether the recovered dynamics of **y** (ŷ(*t*+*l*)|**M_x_**) are unique to the data rather than statistical properties of **x**, we generate surrogates of **x**—surrogates are generated by resampling **x** without replacement. We then compute the cross mapping from the surrogates of **x** to **y**, and in turn compute a 95% quantile from the null distribution of the multiple surrogates.
